# Neuronal Synchronization and Bidirectional Activity Spread Explain Efficient Swimming in a Whole-Body Model of Hydrozoan Jellyfish

**DOI:** 10.1523/JNEUROSCI.1370-24.2025

**Published:** 2025-04-09

**Authors:** Fabian Pallasdies, Philipp Norton, Jan-Hendrik Schleimer, Susanne Schreiber

**Affiliations:** ^1^Department of Biology, Institute for Theoretical Biology, Humboldt-Universität zu Berlin, Berlin 10115, Germany; ^2^Bernstein Center for Computational Neuroscience, Berlin 10115, Germany

**Keywords:** biophysical neuron model, computational fluid dynamics, network model, neural synchronisation, swimming behaviour, whole-body model

## Abstract

Aquatic animals need tightly choreographed movements to efficiently navigate through open waters. Radially symmetric animals, like jellyfish, face the additional challenge of having to respond to regionalized sensory stimuli at the margin of their bell with an orchestrated motor response that initiates predation or escape. The nerve net of hydrozoan jellyfish comprises a condensed ring of electrically coupled neurons, that process sensory input and control the motor output. Here, we aim to understand the coupling of neural activity and motor response by developing a biophysical computational model of the swimming-motor-net of a hydrozoan jellyfish and let it control a swimming jellyfish in a fluid simulation. We find that the neuron activity can synchronize while the signal travels around the ring, eventually triggering a bidirectional wave of activation in the muscles. This mechanism explains seemingly contradicting electrophysiological experiments and minimizes muscle contraction time. Hydrodynamical simulations demonstrate that this setup enables symmetric movement even if neural input is highly asymmetric. We hypothesize that the development of this ring structure supports the jet propulsion by which hydrozoan jellyfish swim. These findings show the importance of considering whole body anatomy and movement when investigating neural design.

## Significance Statement

Hydrozoan jellyfish use simple nerve nets for muscle activation to swim forward by ejecting water with their bell-shaped body. How a directed swimming stroke is created from spatially coordinated activation of their nerve and muscle nets is currently not understood. We demonstrate that these jellyfish use an unexpected strategy involving synchronization of electrical pulses in their nerve net, triggering a parallel spread of activity in different sections of the muscle ring and resulting in a significant reduction of the muscle contraction time. Fluid-dynamical simulations of the whole animal in its aquatic environment show that this clever activation mechanism fosters efficient swimming. The study provides a rare example of a complete mechanistic explanation of animal behavior from cellular biophysics to whole-body movement.

## Introduction

Neural systems have evolved to coordinate and control animal behavior (Jékely et al., 2015). Yet, a mechanistic understanding of the link between neural activity and behavior is not necessarily easy to obtain (Katz, 1996). Belonging to the simplest animals in terms of both anatomy and structural organization of their nervous system, jellyfish (Cnidaria) range among the best suited model organisms to bridge the gap between neural features and behavior. Moreover, their phylogenetic position and evolutionary age (Dunn et al., 2022; Schultz et al., 2023) suggest that their nervous systems can provide insights into the early evolution of neurons and nerve nets. Genetic studies indicate a large divergence in their within-group evolution, with little overlap in jellyfish-specific genes between species (Schnitzler, 2019). On the other hand, their positioning as sister group to the bilaterians with an overlap in many genes enables a comparative view on the evolution of features present in both clades (Moroz and Romanova, 2021; Schultz et al., 2023).

Here, we take advantage of the relative simplicity of the cnidarian medusa bauplan and construct a whole-body computational model of the swimming motor nerve net and associated muscles of a hydrozoan jellyfish. We aim to understand how the electrophysiology and structure of the hydrozoan motor nerve net enable straight and stable swimming motion. As their nervous system belongs to one of the most thoroughly studied ones among hydromedusae, we focus on *Polyorchis penicillatus*. By integrating existing electrophysiological, morphological and behavioral knowledge in this model, we replicate and analyze the full course of events leading up to (and including) an effective swimming stroke, from activity propagation in a network of physiologically detailed neurons and muscle cells to the animal’s mechanical interactions with the fluid environment. The analysis allows us to gain a mechanistic understanding how localized sensory stimuli can result in fast and symmetric activation of all muscles and efficient, directed swimming behavior. Specifically, we gain insights about the relation between synaptic speed and swimming efficiency and resolve a seemingly paradox, negatively correlated relationship between neuron spike width and postsynaptic muscle depolarization in *P. penicillatus*, allowing us to provide an alternative explanation for this phenomenon, which has previously been attributed to a change in synchronization (Spencer et al., 1989).

After outlining the methodological aspects of the study, we start out with background information on the jellyfish nervous system (Katsuki and Greenspan, 2013). We then adapt our recently developed computational model of a scyphozoan nervous system (Pallasdies et al., 2019) to the inner nerve ring and muscles of the hydrozoan *P. penicillatus*, using conductance-based neuron models. Next, we study how the nerve ring with its gap junction-coupled, parallel running fibers can use synchronization in neural activity to produce stable swimming motion. To relate the neurophysiological mechanisms to behavior, we simulate a model of a swimming hydrozoan jellyfish in a 2D fluid environment. We study if, and how, neural synchronization influences swimming performance of hydrozoan jellyfish and identify functional causes for the dichotomy in nerve net design between scyphozoans and hydrozoans.

## Methods

In the following, a whole-body model of *hydrozoan* swimming behavior is introduced. It is composed of a new conductance-based model for neurons in the *hydrozoan* swimming motor net (SMN), a model for muscle dynamics and a spring-based subumbrella model. All of this is embedded into a simplified hydrodynamics simulation to create swimming behavior.

### Code accessibility

Simulations were performed in Python 3.12 using the brian2 package (Stimberg et al., 2019) and code to produce 2D and 3D simulations is available at https://github.com/fpallasdies/HydrozoaNerveNet.

### Neuronmodel

The conductance-based model for the SMN neurons developed and fitted to electrophysiological data is based on Przysiezniak and Spencer (1994) and Grigoriev et al. (1996). The membrane voltage is given by

$$C_{m}\frac{dV}{dt}=I_{\text{syn}}-I_{{\text{Na}}_{\text{fast}}}-I_{{\text{Na}}_{\text{slow}}}-I_{{\text{K}}_{\text{fast}}}-I_{{\text{K}}_{\text{slow}}}-I_{{\text{Ca}}_{\text{fast}}}-I_{{\text{Ca}}_{\text{slow}}}-I_{\text{L}}-I_{\text{Gap}}.\;(1)$$

Individual currents are defined as follows:

$$I_{{\text{Na}}_{\text{fast}}}=g_{{\text{Na}}_{\text{f}}}G_{a}^{\;p_{a}}G_{b}^{\;p_{b}}(V-E_{\text{Na}}),\;(2a)$$

$$I_{{\text{Na}}_{\text{slow}}}=g_{{\text{Na}}_{\text{s}}}G_{c}^{\;p_{c}}G_{d}^{\;p_{d}}(V-E_{\text{Na}}),\;(2b)$$

$$I_{{\text{K}}_{\text{fast}}}=g_{{\text{K}}_{\text{f}}}G_{e}^{p_{e}}G_{f}^{p_{f}}(V-E_{\text{K}}),\;(2c)$$

$$I_{{\text{K}}_{\text{slow}}}=g_{{\text{K}}_{\text{s}}}G_{g}^{\;p_{g}}(V-E_{\text{K}}),\;(2d)$$

$$I_{{\text{Ca}}_{\text{fast}}}=g_{{\text{Ca}}_{\text{f}}}G_{h}^{p_{h}}G_{i}^{p_{i}}(V-E_{\text{Ca}}),\;(2e)$$

$$I_{{\text{Ca}}_{\text{slow}}}=g_{{\text{Ca}}_{\text{s}}}G_{\;j}^{\;p_{j}}(V-E_{\text{Ca}}),\;(2f)$$

$$I_{\text{L}}=g_{\text{L}}(V-E_{\text{L}}),\;(2g)$$

$$I_{\text{Gap}}=\sum_{i}g_{{\text{gap}}_{i}}(V-V_{i}),\;(2h)$$

$$I_{\text{syn}}=g_{\text{\{syn\}}}\;(E_{\text{syn}}-v),\;(2i)$$

$$\frac{\text{d}}{\text{d}t}g_{\text{syn}}=-g_{\text{syn}}/\tau_{\text{syn}}.\;(2j)$$

The gate variable *G*_*i*_ with i∈{a,…,g} is given by

$$\tau_{i}\frac{dG_{i}}{dt}=G_{i\infty }(V)-G_{i},\;(3)$$

with

$$G_{i\infty }(V)=\frac{1}{1+\exp\;\frac{V_{1/2_{i}}-V}{\rho_{i}}}.\;(4)$$

The time scale variables *τ*_*i*_ with i∈{a,…,g} are given by

$$\tau_{i}(V)=C_{{\text{base}}_{i}}+C_{{\text{amp}}_{i}}\exp\;\left(\frac{-(V_{{\text{max}}_{i}}-V{)}^{2}}{\sigma_{i}^{2}}\right).\;(5)$$

The calcium current was modelled with a slow and fast channel, similarly to the sodium and potassium channels. We found its influence on neuronal dynamics to be limited, as the resulting calcium current is several magnitudes smaller than the potassium current. The neuron ring was modelled as a set of electrically coupled compartments; coupling strength *g*_gap_ between neuron compartments was determined by the cytoplasmic resistance 56 Ω/cm (Spencer, 1981). Scyphozoan synapses are bidirectional (Horridge and Mackay, 1962; Anderson, 1985) and scyphozoan neurons do not differentiate between axons and dendrites. Similarly hydrozoan SMN neurons do not show directionality in morphology or in electrophysiological experiments (Arkett and Spencer, 1986a; Lin et al., 2001). Therefore,neurons were modelled as bidirectional, homogeneous strips of active cable.

**Figure 1.**
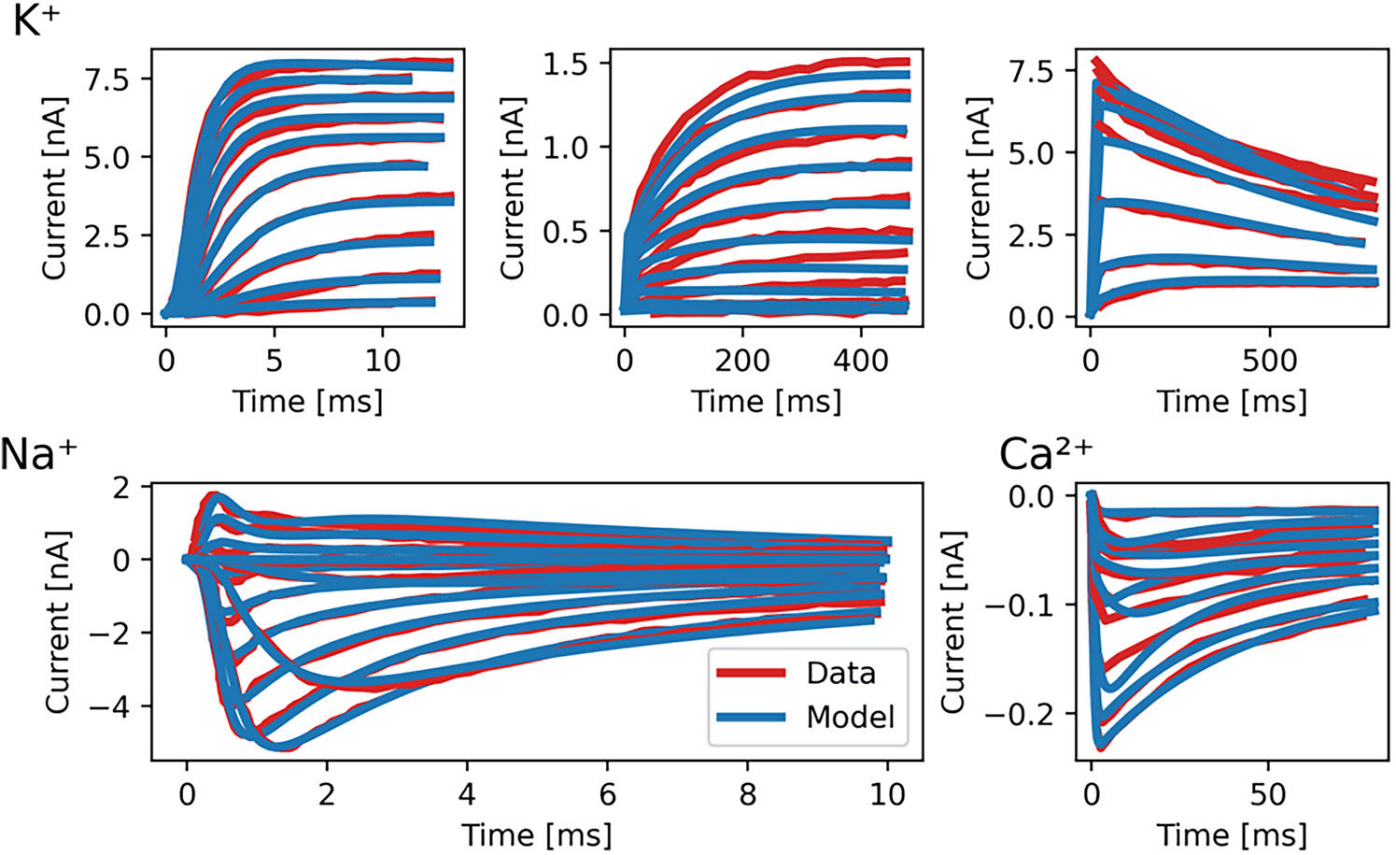
The fit of K^+^, Na^+^ and Ca^2+^ ion channel models on voltage-clamp data. A comparison between experimental voltage-clamp recordings of SMN neurons with conductance based models for the different ion channels. Data for these experiments were extracted from Przysiezniak and Spencer (1994) in Figure 2, 3 & 4, Grigoriev et al. (1996) in Figure 1 and Przysiezniak and Spencer (1992) in Figure 5.

Parameters were fitted by first extracting the results of voltage clamp experiments (Przysiezniak and Spencer, 1992, 1994; Grigoriev et al., 1996) using the WebPlotDigitizer (Rohatgi, 2019). Then we fitted the parameters in batches of the relevant ion channels studied, i.e., Ca, Na and K channels. Parameters were fitted using the modified Powell algorithm using the SciPy package (Powell, 1964; Jones et al., 2001). To circumvent local minima, the basin hopping algorithm was applied to rerun the optimization several times from different starting points until no further improvements were found (Olson et al., 2012). Fit quality can be assessed in Figure 1, showcasing experimental and model traces. Optimized parameters are stated in Table 1.

**Table 1.** Neuron model parameters

Variable	Value	Unit	Description
*C*_*m*_	1.52	μF/cm^2^	Capacitance (Spencer, 1981)
$g_{{\text{Na}}_{\text{fast}}}$	13.8	mS/cm^2^	Peak conductance
$g_{{\text{Na}}_{\text{slow}}}$	3.5	mS/cm^2^	
$g_{{\text{K}}_{\text{fast}}}$	6.27	mS/cm^2^	
$g_{{\text{K}}_{\text{slow}}}$	1.15	mS/cm^2^	
$g_{{\text{Ca}}_{\text{fast}}}$	0.075	mS/cm^2^	
$g_{{\text{Ca}}_{\text{slow}}}$	0.038	mS/cm^2^	
*g*_L_	0.038	mS/cm^2^	
$g_{{\text{gap}}_{\text{circular}}}$	27.99	mS/cm^2^	
$g_{{\text{gap}}_{\text{radial}}}$	5.6	mS/cm^2^	
*E*_Na_	54.15	mV	Reversal potential
*E*_K_	−60.75	mV	
*E*_Ca_	90.84	mV	
*E*_L_	−60	mV	
*E*_syn_	54.15	mV	
*τ*_syn_	25	ms	Synaptic time constant
*p*_*a*_	7.57		Gating exponent
*p*_*b*_	1.65		
*p*_*c*_	3.96		
*p*_*d*_	0.78		
*p*_*e*_	2.89		
*p*_*f*_	1.93		
*p*_*g*_	1		
*p*_*h*_	1		
*p*_*i*_	1.13		
*p*_*j*_	5.44		
$V_{{1/2}_{a}}$	−16.5	mV	Half-maximal voltage
$V_{{1/2}_{b}}$	−18.58	mV	
$V_{{1/2}_{c}}$	−21.5	mV	
$V_{{1/2}_{d}}$	−30.65	mV	
$V_{{1/2}_{e}}$	−21.54	mV	
$V_{{1/2}_{f}}$	−43.33	mV	
$V_{{1/2}_{g}}$	34.54	mV	
$V_{{1/2}_{h}}$	−4.5	mV	
$V_{{1/2}_{i}}$	−17.82	mV	
$V_{{1/2}_{j}}$	−20.39	mV	
*ρ*_*a*_	4.98	mV	Slope of gating
*ρ*_*b*_	−9.06	mV	voltage dependence
*ρ*_*c*_	2.76	mV	
*ρ*_*d*_	−6.24	mV	
*ρ*_*e*_	13.22	mV	
*ρ*_*f*_	−9.47	mV	
*ρ*_*g*_	19.28	mV	
*ρ*_*h*_	5.5	mV	
*ρ*_*i*_	−1	mV	
*ρ*_*j*_	5.15	mV	
$C_{{\text{base}}_{a}}$	0.14	ms	Base time constant
$C_{{\text{base}}_{b}}$	0.6	ms	
$C_{{\text{base}}_{c}}$	0.72	ms	
$C_{{\text{base}}_{d}}$	6.62	ms	
$C_{{\text{base}}_{e}}$	0.85	ms	
$C_{{\text{base}}_{f}}$	31.34	ms	
$C_{{\text{base}}_{g}}$	121.73	ms	
$C_{{\text{base}}_{h}}$	0.33	ms	
$C_{{\text{base}}_{i}}$	24.94	ms	
$C_{{\text{base}}_{j}}$	0.26	ms	
$C_{{\text{amp}}_{a}}$	0.33	ms	Determines peak
$C_{{\text{amp}}_{b}}$	1.35	ms	gating time constant
$C_{{\text{amp}}_{c}}$	1.04	ms	value
$C_{{\text{amp}}_{d}}$	648	ms	
$C_{{\text{amp}}_{e}}$	1.93	ms	
$C_{{\text{amp}}_{f}}$	2.1	s	
$C_{{\text{amp}}_{g}}$	637.19	ms	
$C_{{\text{amp}}_{h}}$	46.83	ms	
$C_{{\text{amp}}_{i}}$	43.35	ms	
$C_{{\text{amp}}_{j}}$	1.51	ms	
$V_{{\text{max}}_{a}}$	−33.23	mV	Determines peak
$V_{{\text{max}}_{b}}$	−12.88	mV	gating time constant
$V_{{\text{max}}_{c}}$	−63.75	mV	voltage
$V_{{\text{max}}_{d}}$	−31.62	mV	
$V_{{\text{max}}_{e}}$	−17.1	mV	
$V_{{\text{max}}_{f}}$	32.563	mV	
$V_{{\text{max}}_{g}}$	−29.56	mV	
$V_{{\text{max}}_{h}}$	47.9	mV	
$V_{{\text{max}}_{i}}$	5.68	mV	
$V_{{\text{max}}_{j}}$	−54.51	mV	
*σ*_*a*_	36.7	mV	Width of the gaussian
*σ*_*b*_	56	mV	determining the gating
*σ*_*c*_	14.69	mV	time constant
*σ*_*d*_	5.46	mV	
*σ*_*e*_	41.58	mV	
*σ*_*f*_	29.98	mV	
*σ*_*g*_	19.27	mV	
*σ*_*h*_	15.52	mV	
*σ*_*i*_	5.26	mV	
*σ*_*j*_	41.65	mV	

### Muscle dynamics

To simulate the dynamics of the epitheliomuscular cells, we constructed a Morris-Lecar model (Morris and Lecar, 1981). Since there is not enough electrophysiological data available to try to fit the model, we modified the parameters manually, such that the length of a muscle spikes is in agreement with measurments in *P. penicillatus* (Spencer and Satterlie, 1981; Spencer, 1982; Satterlie, 2015). The dynamics of the muscle voltage are given by

$$C_{m}\frac{dV}{dt}=I_{\text{syn}}-g_{\text{L}}\left(V-E_{\text{L}}\right)-g_{\text{K}}N\left(V-E_{\text{K}}\right)-g_{\text{Ca}}M_{\infty }\left(V-E_{\text{Ca}}\right)-I_{\text{Gap}},\;(6a)$$

$$\frac{dN}{dt}=\frac{N_{\infty }-N}{\tau_{N}},\;(6b)$$

where *I*_Gap_ is defined in the same manner as Equation 2af, *N*_∞_ and *M*_∞_ are following Equation 4 and *τ*_*N*_ is following Equation 5. These muscle cells cover almost the whole subumbrella except for the manubrium, which is enclosed by the smaller ring. Muscle cells are electrically coupled to their neighbors, where coupling strength circularly is much stronger than radially (see Table 2). The synaptic currents of the muscle cells created by presynaptic neurons is given by a simple exponentially decaying conductance, increased by a delta function with:

$$I_{\text{syn}}=g_{\text{syn}}h(E_{\text{syn}}-V)\;(7a)$$

$$\frac{dh}{dt}=\sum_{i}\delta (t-t_{i})-h/\tau_{h},\;(7b)$$

where *δ* is the Dirac delta function where *t*_*i*_ is the timepoint a presynaptic neuron spikes and *τ*_*h*_ is the time constant of the synapse.

**Table 2.** Muscle model parameters

Variable	Value	Unit	Description
*C*_*m*_	1	μF/cm^2^	Capacitance
*g*_K_	6.3	mS/cm^2^	Peak conductance
*g*_Ca_	13.5	mS/cm^2^	
*g*_syn_	57.59	μS/cm^2^	
*g*_L_	0.75	mS/cm^2^	
$g_{{\text{gap}}_{\text{circular}}}$	1.17	mS/cm^2^	
$g_{{\text{gap}}_{\text{radial}}}$	0.234	mS/cm^2^	
*E*_K_	−90	mV	Reversal potential
*E*_Ca_	60	mV	
*E*_L_	−83.5	mV	
*E*_syn_	60	mV	
$V_{{1/2}_{N}}$	−15	mV	Half-maximal voltage
$V_{{1/2}_{M}}$	−8	mV	
*ρ*_*N*_	3.5	mV	Slope of gating voltage dependence
*ρ*_*M*_	10	mV	
*τ*_*h*_	3	ms	Synaptic time constant
$C_{{\text{base}}_{N}}$	50	ms	Base time constant
$C_{{\text{amp}}_{N}}$	92	ms	Determines peak time constant
$V_{{\text{max}}_{N}}$	10	mV	Voltage of peak time constant
*σ*_*N*_	18	mV	Width of the Gaussian describing the time constant

### 2D model subumbrella

**Figure 2.**
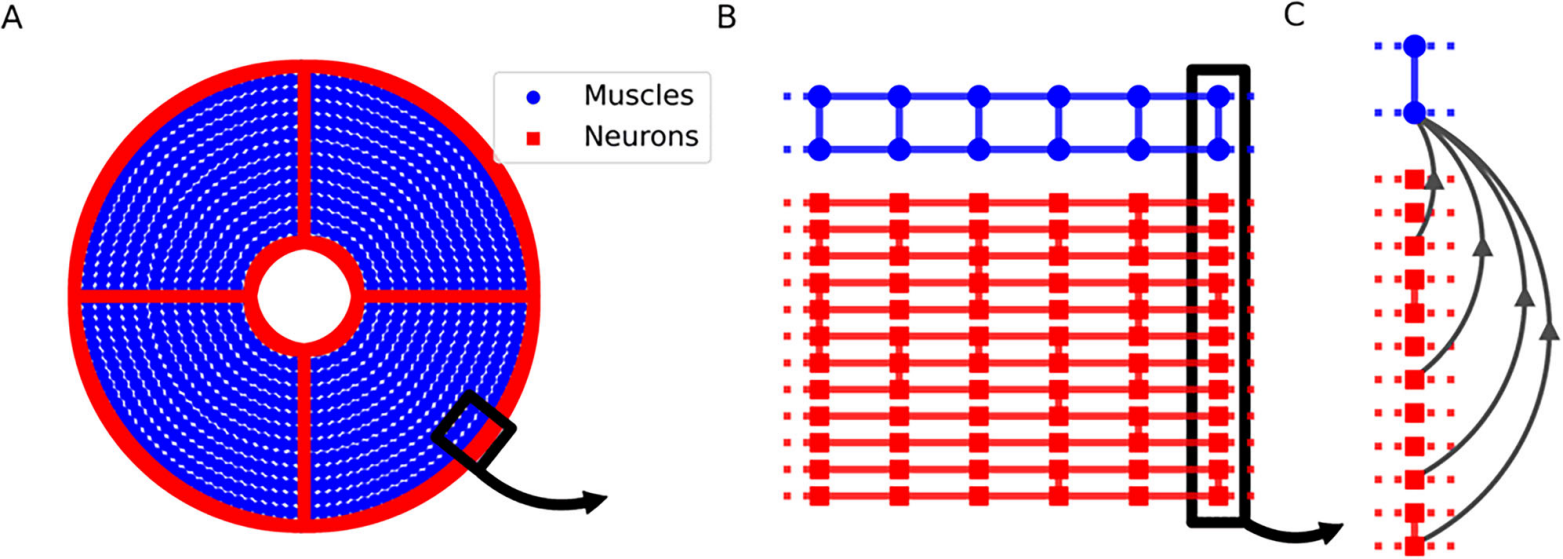
Simplified illustration of the model setup. ***A***, Overview of how the neurons and muscles are set up. SMN neurons (red) are arranged in two rings, one at the margin of the bell and one at the center with a radius corresponding to 1/4th of the total bell radius. These rings are connected via 4 radial canals. Muscles (blue) are arranged in concentric circles between the margin of the bell and the manubrium. ***B***, Zooming in, neurons belong to one of twelve parallel running fibers that are interconnected as shown with a probability of 0.3. Muscles are connected in a lattice pattern both circularly and radially. In both cases these connections are based on electrical junctions and radial connections are weaker than circular connections. ***C***, Neurons are connected to the closest muscle cell via chemical synapses with a probability of 0.25.

To model the interaction of muscles and SMN neurons on the whole subumbrella, we built a model of the inner nerve ring, the neurons in the four radial canals and the the connections between them. An overview of this setup can be seen in Figure 2*A*. We model the inner nerve ring of *P. penicillatus* as a set of rings with random connections between them (see Fig. 2*B*). Within a ring each neuron compartment is electrically coupled to both of its neighbors (*g*_*c*_) while connections between the rings (*g*_*r*_) are randomly placed with the probability *p*_*r*_ = 0.3 between neighboring neurons of different rings, creating a lattice structure. Neurons in the radial canal are organized in the same way as in the marginal nerve ring, with the same number of parallel running fibers and a 30 % probability that a given compartment in one fiber is coupled with a neighboring parallel running compartment. In all simulations shown, we used a set of twelve neuron rings where each neuron/segment has a length of 175 μm. In the 2D model of the subumbrella with a radius of 1.5cm, this results in 6,480 neurons in the marginal ring and 11,136 neurons on the whole subumbrella. Muscle compartments are modeled as squares with a side length of 175 μm resulting in a total of 22,204 muscle segments simulated in the subumbrella model.

Every neuron has a probability *p*_syn_ = 0.25 to be presynaptic to it’s closest neighboring muscle cell (Fig. 2*C*), which are arranged in concentric circles along the subumbrella and coupled to their neighbors. The coupling in the circular direction however is five times larger than the radial coupling, to create the differences in conduction speed seen in real *P. penicillatus* muscles (Spencer, 1978, 1982).

### Hydrodynamics

To simulate the swimming motion, we use the IB2D package (Battista et al., 2015, 2017a, 2017b) designed for the creation of simple 2D hydrodynamics simulations with fluid-structure interaction. This package simulates fluid-structure interactions using the immersed boundary method (Peskin, 1972, 2002) by simulating the fluid on a grid, and the structure (i.e., the jellyfish) as a set of vertices with mechanical springs between them. In each simulation step, one alternates between (1) calculating the flow of the fluid and its forces acting onto the vertices of the structure and (2) moving the vertices according to their summed forces and calculating the influence of the vertices onto the fluid mesh. The details of the simulations presented here mostly follow the methods of Pallasdies et al. (2019). Nevertheless, a comprehensive description of the simulation setup follows.

The structure of the jellyfish bell is determined by a set of vertices that are connected via mechanical springs, to create a cross section of the umbrella. The subumbrellar vertices are equidistantly placed, starting from the center of the jellyfish, where the position of the following vertex *i* is determined by an angle ϕ given by

$$\varphi (i)=\alpha \cdot \min\left[{\left(\frac{i}{m\cdot N_{p}}\right)}^{n},1\right],\;(8)$$

where *N*_*p*_ is the total number of vertices used to construct one half of the subumbrella and *α* is the maximum angle. If *α* = 0, then the subumbrella of the model is just a flat horizontal line, then *α* = *π*/2 means that the margins of the subumbrella will point straight down.

The vertices on the exumbrella of the cross section are always placed orthogonal to the subumbrella, where

$$h(i)=C_{\text{base}}(N_{p}-i)+C_{\text{amp}}\exp\;\left(\frac{i^{2}}{\sigma^{2}}\right),\;(9)$$

describes the distance to the subumbrella. Here, *C*_base_ is the minimum thickness of the umbrella.

The vertices along the exumbrella and sumbumbrella are connected via a set of springs and the two epithelia a connected via a cross hatching pattern with springs (see Pallasdies et al., 2019). These create forces on the vertices that creates the motion of the jellyfish bell. If two vertices with the positions *X*_1_ and *X*_2_ are connected via a spring with resting length *R*_*L*_ and stiffness *k*_*s*_, then the force on these vertices at any given time is described by

$$F_{\text{s}}=k_{s}\left(1-\frac{R_{L}}{\left\|X_{1}-X_{2}\right\|}\right)\left(X_{1}-X_{2}\right)+b_{s}\frac{\text{d}}{\text{d}t}\|X_{1}-X_{2}\|.\;(10)$$

In our model, muscles are placed from the vertices on the subumbrella to a vertical line at the center of the jellyfish. The resulting force $F_{m_{i}}$ is given by dividing the subumbrella in 64 sections and summing over the voltages *V*_*j*_ of all muscle cells in the given section. This sum is then normalized such that it varies between 0 and 1. By addition of a force-length relationship, the force action on a particular vertex is given by

$$F_{m_{i}}=F_{\text{max}}\frac{\sum_{j}^{n_{i}}\max\left(V_{j}+10\text{mV},0\right)}{V_{\text{max}}}\exp\;\left[-{\left(\frac{L_{F}^{i}/L_{O}^{i}-1}{S}\right)}^{2}\right].\;(11)$$

Here, $L_{F}^{i}$ and $L_{O}^{i}$ are the current length and the length of the muscle at rest, respectively, *F*_max_ is the maximum muscle force and *V*_max_ is the normalization of the sum of muscle voltages i.e., the maximum voltage of all muscles in a given segment such that the values never exceed *F*_max_. All relevant parameters of the fluid simulation can be found in Table 3.

**Table 3.** Hydrodynamics simulation parameters

Variable	Value	Unit	Description
*α*	1.1*π*/2		Maximum angle
*m*	0.91		Shape parameter
*N*_*p*_	420		Number of vertices on a subumbrella half
*n*	0.6		Shape parameter
*C*_base_	1.6	mm	Minimum subumbrella thickness
*C*_amp_	1	cm	Determines maximum thickness
*σ*	316.23		Determines width distribution along the subumbrella
$k_{s}^{\text{surface}}$	1 · 10^8^	N/m	External spring stiffness
$k_{s}^{\text{internal}}$	4 · 10^8^	N/m	Internal spring stiffness
*b*_*s*_	2.5	kg/s	Damping coefficient
μ	0.005	Ns/m^2^	Fluid density
*ρ*	1000	kg/m^2^	Fluid viscosity
Time step	10^−5^	s	
*x*-length of Eulerian grid	0.08	m	
*y*-length of Eulerian grid	0.16	m	
*x*-grid size	150		
*y*-grid size	300		
S	0.35		Muscle length sensitivity
*F*_Max_	0.5	N	Maximum muscle force

## Results

### Background on the jellyfish nervous system

**Figure 3.**
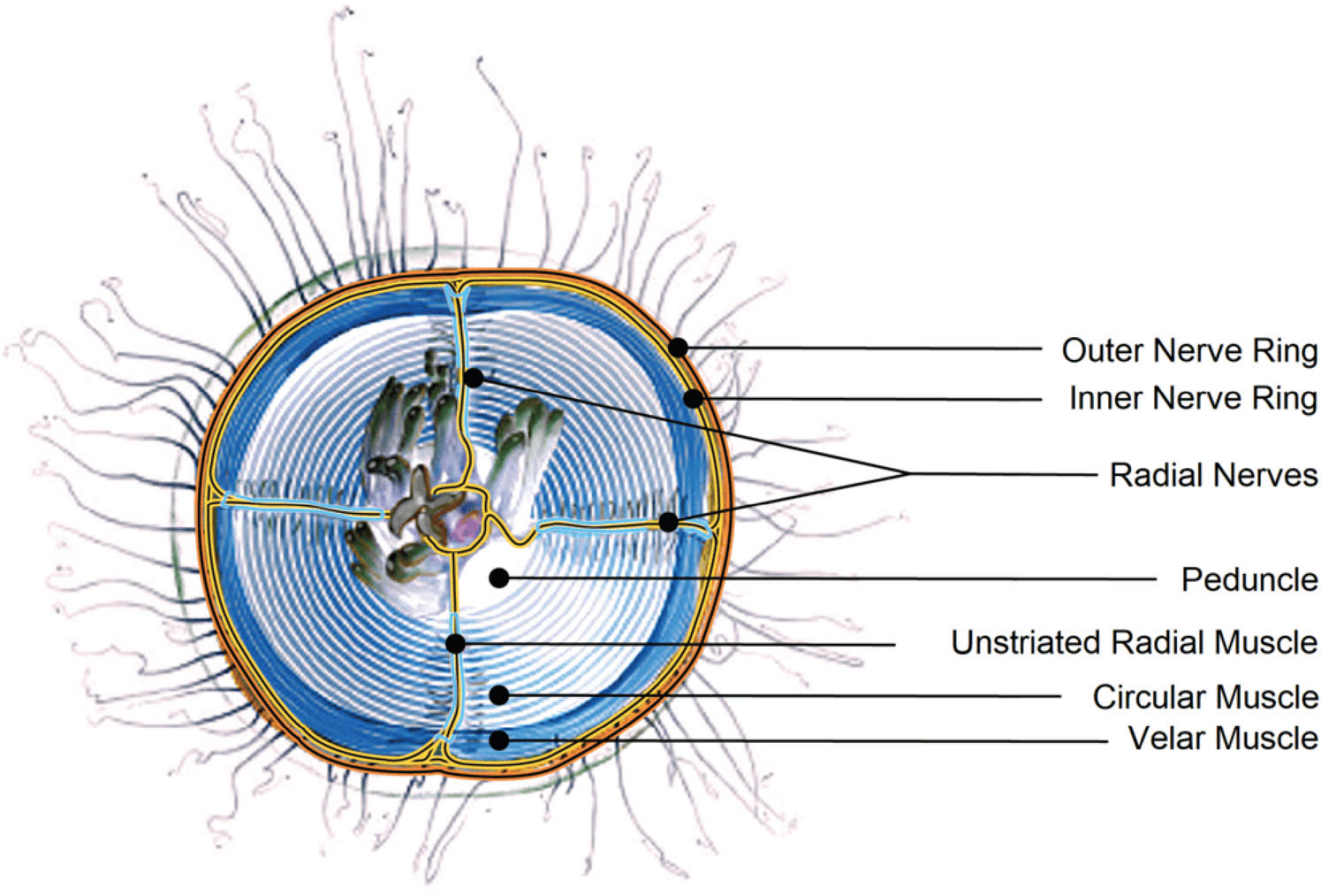
Illustration of the anatomy of *P. penicillatus*. A schematic of the ventral view on *P. penicillatus*. Shown is the sumbumbrella (the underside of the bell) with both muscles and neurons highlighted. Neurons are located in the nerve rings on the margin of the bell and the radial canals (shown here as radial nerves). The swimming motor net (SMN) is restricted to the inner nerve ring and the radial canals and innervates the circular swimming muscles. Credits to Emily Lowes (permission obtained).

While swimming jellyfish medusae seem to have a relatively small behavioral repertoire, they exhibit huge differences in the structure of their nervous systems (Satterlie, 2002). Scyphozoan jellyfish (or *true jellies*) generally have diffusely distributed nerve nets connected through chemical synapses, covering the subumbrella (the mouthwards side of the animal) or the whole bell (Schäfer, 1878; Passano and Passano, 1971; Mackie, 1980; Mackie et al., 1984). In contrast, hydrozoan jellyfish have a set of nerve rings along their bell margin (two in *P. penicillatus*: inner and outer nerve ring; Fig. 3) with which they control their movement (Singla, 1978; Romanes, 1885; Lin et al., 2001; Satterlie, 2002). Out of those, the neurons of the inner nerve ring, as well as of the four radial canals, are responsible for activating the circular muscles in the subumbrella which ultimately produce the swimming motion (Anderson and Mackie, 1977; Spencer and Arkett, 1984). These neurons are hence commonly referred to as “swimming motor net” (SMN) (Spencer, 1982; Satterlie, 2002).

The SMN in *P. penicillatus* comprises a dense network of relatively big but short bipolar neurons with little branching (Spencer, 1981; Przysiezniak and Spencer, 1992), electrically coupled via gap junctions (Mackie, 1980; Spencer and Satterlie, 1980). SMN neurons also form chemical synapses with their neighbouring epithelial cells that conduct the signal to the circularly oriented epitheliomuscular cells in the underside of the bell (Spencer, 1978; Satterlie and Spencer, 1983). When a set of SMN neurons gets experimentally stimulated, a wave of single spikes travels around the ring and up the four radial canals that segment *P. penicillatus* into four quadrants (Spencer, 1982; Lin et al., 2001). This mechanism leads to a contraction of the circular, striated muscles (Fig. 3) mainly responsible for the swimming motion (Anderson and Mackie, 1977; Spencer, 1981, 1982).

The most discussed feature of the SMN electrophysiology is the variability in action potential spike width. The depolarization via action potential of an SMN neuron can vary between 8 and 50 ms in *P. penicillatus* (Anderson, 1979; Spencer, 1981, 1982). Multi-electrode experiments show that the width of these spikes is not random. Neurons close to the stimulus site consistently show wider spikes than neurons further away, with very little variation in the amplitude of these spikes (Spencer, 1982). In experiments, a strong negative correlation between neuron spike width and muscle depolarization has been measured (Spencer, 1982). Very little research has aimed to explain this paradoxical behavior and its purpose so far, but Spencer (1982) proposed that the muscle response might arise due to a change of synchrony of the muscle’s innervating neurons, and that this change in synchrony might cause the varying spike widths. In order to shed light on the underlying mechanism, we first establish a mathematical model of the SMN.

### Spike shortening depends on potassium channel inactivation

**Figure 4.**
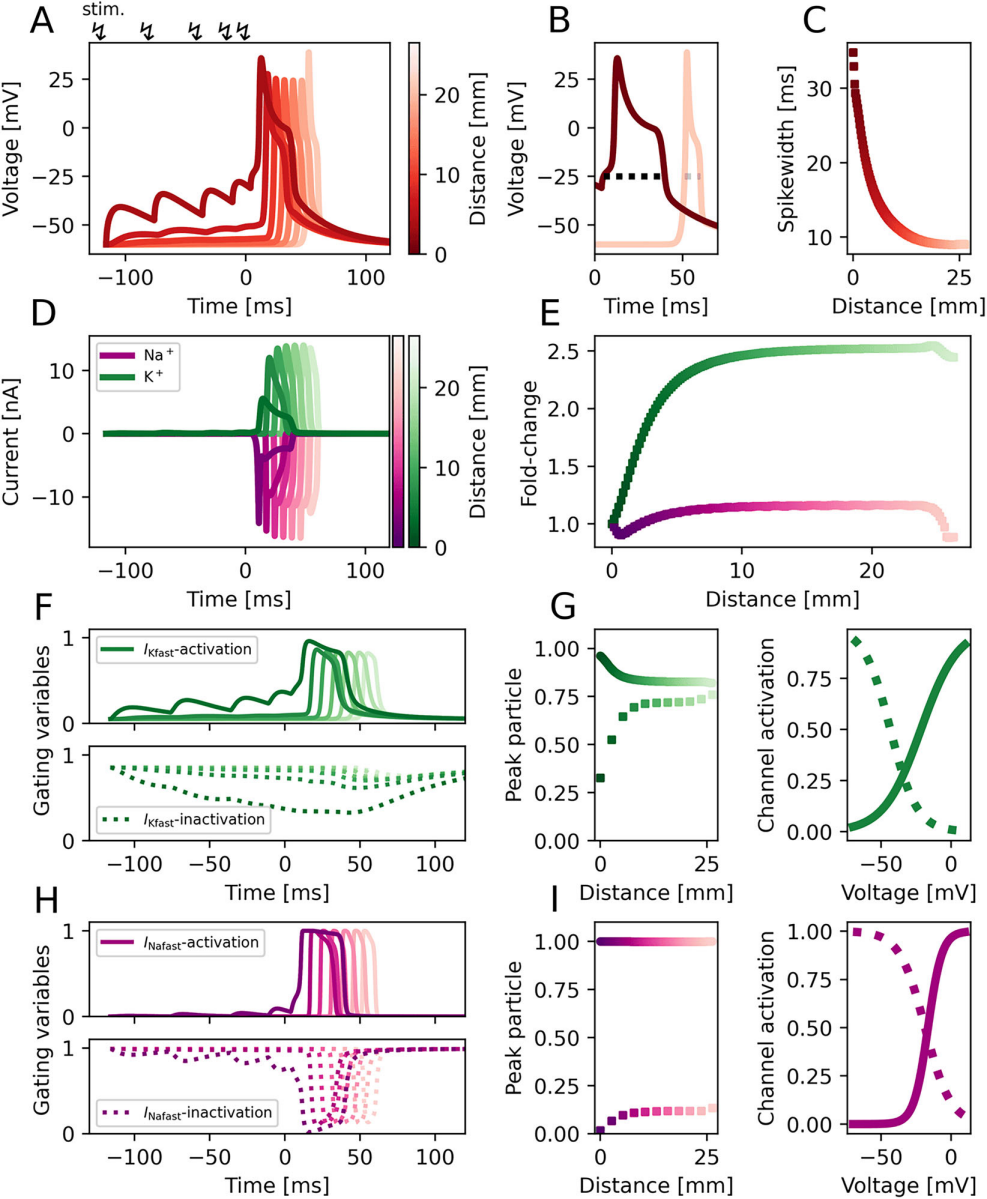
Spike shortening in a strip of SMN neurons is caused by potassium inactivation. Traces of a strip of electrically coupled SMN neurons excited by several synaptic pulses (.55 mS/cm^2^ in strength) on one end of the strip until a spike is elicited that travels along the entire strip (2.65 cm in length). Brightness of traces indicates the distance from the site of stimulation at one end of the strip throughout the whole figure (increasing with distance). Colorbars therefore essentially represent the neuron strip, with the stimulation site at the dark end of it ***A***, The voltage measured in different neurons along the strip. ***B***, Spike width at the beginning and end of the neuron strip, respectively. To measure spike width, a voltage threshold of −25 mV was used. ***C***, Neuronal spike width as a function of location on the strip. Wider spikes are found closer to the site of spike initiation. ***D***, Ionic currents evoked by potassium and sodium channels. ***E***, Fold-change of the absolute peak currents along the strip (for sodium and potassium, respectively). ***F***, Gating variables for the activation and inactivation of the fast potassium channel. ***G***, Minimum and maximum of the activating and inactivating gating variables, respectively along the strip (left) and activation curves of the gating variables (right). ***H*** & ***I***, Description of the sodium channel gating variables analogous to *F* & *G*. Calcium currents can be seen in Extended Data Fig. 4-1.

We quantitatively fitted a conductance-based neuron model to reproduce experimental voltage-clamp data of SMN neurons (Przysiezniak and Spencer, 1992, 1994; Grigoriev et al., 1996) with high fidelity (for details see Methods). This model consists of two transient sodium channels and a transient and a steady state potassium channel (see Eq. 1 for details). We also fitted two calcium channels to corresponding voltage clamp data but due to the orders of magnitute smaller currents it does not influence the behavior of the model. Due to the timescale of spiking the neuron behavior mainly depends on the fast transient sodium and potassium channels, on which we will focus on for the following analysis. This fitted model allows us to uncover potential mechanisms underlying the neuronal control of hydrozoan swimming behavior. We first turn toward one of the most studied phenomena in *P. penicillatus* electrophysiology – the variability of spike widths. Measured between 8 and 40 ms, this variability has originally been attributed to synchronous firing of SMN neurons (Anderson and Mackie, 1977) and described as a side effect of the electrical coupling between them. Anderson (Anderson, 1979) first saw that, counter-intuitively, strong input currents create rather short spikes while weak input currents create long spikes after a significant delay and concluded that the level of depolarization prior to a spike determines the width of a spike. Spencer et al. (1989) proposed that the difference in spike width might depend on the inactivation of the fast potassium current due to different levels of depolarization of the SMN neurons. To test this hypothesis, we simulate a strip of SMN neurons coupled to each other. Experimentally, this would represent severing the inner nerve ring from the jellyfish and cutting the ring at one point to get a long strip simulated here as a chain of SMN neurons, each connected to their neighbors. Because of the electric coupling between the neurons, this model is essentially a strip of active cable. Stimulation of the neuron on one end of the strip leads to spiking activity that propagates through the entire SMN. We simulate the bursting input from the outer nerve net (likely responsible for the processing of sensory input) by providing multiple pulses to the first neuron of the strip (Spencer and Arkett, 1984). These pulses then drive the other neurons along the strip and are chosen as the appropriate stimulation for the SMN to imitate the behavior of the ’B system’, a set of bursting neurons in the outer nerve ring presynaptic to the SMN that elicit SMN firing (Spencer and Arkett, 1984). Due to the different levels of depolarization, however, the spike width differs between neurons (Fig. 4*A*). The first neuron with the highest depolarization also exhibits the widest spike, while the amplitude of depolarizations progressively decreases due to attenuation along the strip (Fig. 4*B*), accompanied by a reduction in spike width. Spike widths range from 35 to down to 8 ms in very good agreement with the experimentally observed values (Fig. 4*C*, compare with Spencer, 1981, 1982; Przysiezniak and Spencer, 1989).

10.1523/JNEUROSCI.1370-24.2025.f4-1 Figure 4-1 S**p**ike **shortening in a strip of SMN neurons does not meaningfully change calcium current levels.** The simulation setup is identical to Fig. 4 in the main text but here calcium currents are displayed. *Top*: The voltage measured in different neurons along the strip. *Middle*: Ionic currents evoked by calcium channels. *Bottom*: Fold-change of the absolute peak calcium currents along the strip compared to the fold-change of sodium and potassium currents. Similar to sodium, the calcium currents do not meaningfully change along the strip with spike shortening. Download Figure 4-1, TIF file.

Analysis of individual currents (Fig. 4*D*) suggests that this effect mainly arises from changes in the potassium current, because its amplitude increases up to 2.5-fold over the course of the activation wave (Fig. 4*E*). The cause of this change is the inactivation of the fast potassium channel due to the depolarization prior to the spike. This can be seen in the action of both the potassium inactivating and activating particles (Fig. 4*F*). The course of the inactivating particle changes considerably depending on the distance from the stimulation site (Fig. 4*G*). As a result of the different onsets of the inactivation particles (Fig. 4*G*, *right*), the K^+^ channel inactivates due to the depolarization prior to a spike. In contrast, the sodium gating dynamics vary little (Fig. 4*H*). The peak values of the Na^+^ gating particles are largely unaffected because the low voltages of the pre-spike depolarization lie below the onset of their activation curves (Fig. 4*I*). Therefore, the voltage of the depolarized neurons remains at a plateau for a longer period of time after spiking. Only when the Na^+^ channel sufficiently deactivates after spiking, does the voltage return to the resting level. These results provide a holistic explanation for the experimental observations (Anderson, 1979; Spencer et al., 1989) on SMN neurons.

It has been postulated that the spike shortening is essential for the increase of neuromuscular PSPs during a propagation wave, as shorter spikes correlate with stronger PSPs. It has been suggested that shorter spikes increase peak Ca^2+^ currents, resulting in larger PSCs (Anderson and Spencer, 1989; Spencer et al., 1989). In our simulations, however, peak Ca^2+^ currents changed only marginally in relation to spike width, and wider spikes actually have a higher integrated current over time than shorter spikes (see Extended Data Fig. 4-1). While a current-strength-dependent transmitter release could thus, in principle, contribute to a relation between PSP amplitude and spike width, the synaptic activation function would have to be sensitive to very small differences in presynaptic Ca^2+^ if it should be assumed to be the core contributor. Such a high-gain scenario would render synaptic transmission highly sensitive to noise, decreasing the robustness of the PSP spike-width correlation. Therefore we turn to study the muscle activation via the SMN.

### Muscle postsynaptic potentials (PSPs) vary with neural synchronization

To understand the neuromuscular interactions of the SMN, we next investigate a simplified ring model of SMN neurons with a thin layer of muscles connected to it. We use this model to explore the effects of spike shortening on the activity of muscle cells and to replicate the experimental results on ring sections of *P. penicillatus* (Spencer, 1982).

**Figure 5.**
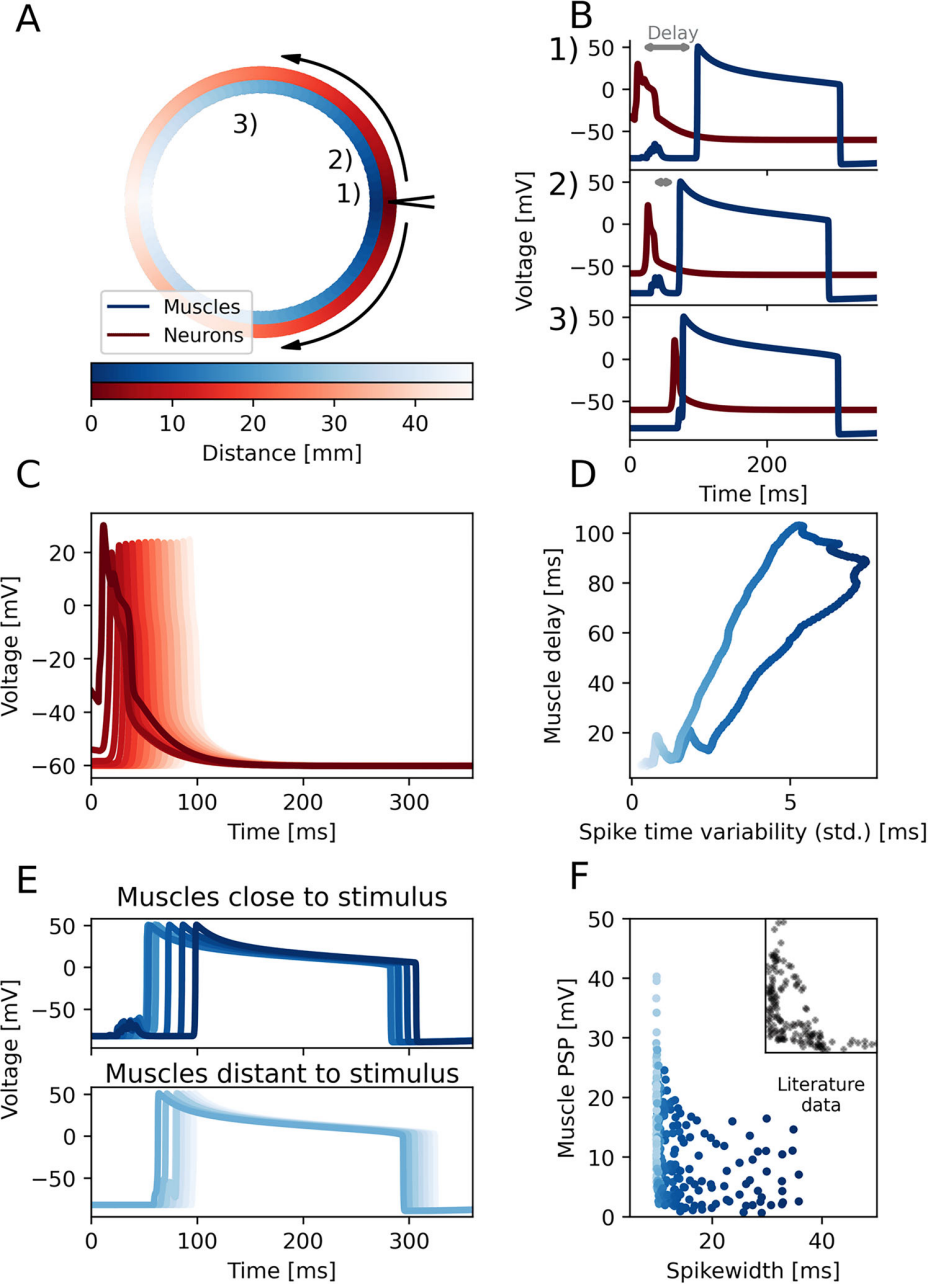
Synchronization of parallel running neuron fibers explains the relation between neuron spike widths and muscle PSPs in *P. penicillatus*. ***A***, An overview of the ring model of the section similar to Spencer (1982); SMN neurons (red) and epitehliomuscular cells (blue). The site of spike initiation is on the very right and the neuron signal conducts through the entire SMN along both sides. Numbers mark the recording sites of traces in *B*. ***B***, Voltage traces of neurons and muscles at three different points along the ring. Grey bars indicate the delay between neuron and muscle spike at the respective location. ***C***, Neuronal voltage traces along the ring; Brightness indicates the distance to the site of initiation. ***D***, Spike time variability of the neurons at a local section of the ring plotted against the delay between the local neuron and muscle spikes measured at both paths along the ring. ***E***, Muscle voltage traces for different positions along the ring, similar to *C*; for better distinguishability muscles grouped by distance to stimulation site (proximal top, distal bottom). ***F***, Peak of subthreshold PSP across muscles cells (deviation from the resting potential) plotted against spike width of a random neuron in the nearest ring segment. Distance between muscle and neuron was always below 60 μm. Inset shows literature data from Spencer (1982). The inset displays data over the same axis range as the main plot. A replication of these results in a simulation without spike shortening can be seen in Extended Data Fig. 5-1.

In those experiments, Spencer (Spencer, 1982) excised a circular section of *P. penicillatus*, leaving only the nerve ring, the velum and a small annulus of the bell (see Fig. 5*A*). This fragment of the jellyfish bell is still able to spike and contract. Shining a light at a section of the ring causes SMN neurons to spike at this location. As the signal travels around the ring, they stimulate nearby epitheliomuscular cells. Both neurons and muscles were previously recorded, revealing the inverse correlation between neuron spike width and muscle PSPs mentioned above. Here, our model reproduces this relation, validating the model and allowing us to understand the underlying spike-shortening mechanism and its effects on muscle activation patterns.

10.1523/JNEUROSCI.1370-24.2025.f5-1 Figure 5-1 **Spike shortening is not necessary for synchronization-based spiking.** Similar model setup to Fig. 5 but the SMN is stimulated via a singular, short input current, which in effect does not produce wide spikes. This destroys the anticorrelation between spike widths and muscle PSPs but retains all other features of our model, including the pattern of muscle activation and the correlation between spike time variability and muscle delay, thus demonstrating that spike shortening is not causal to the varying muscle PSPs in our model. *A*: An overview of the ring model. *B*: Voltage traces of neurons and muscles at three different points along the ring. Grey bars indicate the delay between neuron and muscle spike at the respective location. *C*: Neuronal voltage traces along the ring; brightness indicates the distance to the site of initiation. *D*: Spike time variability of the neurons at a local section of the ring plotted against the delay between the local neuron and muscle spikes measured at both paths along the ring. *E*: Muscle voltage traces for different positions along the ring, similar to *C*; for better distinguishability muscles grouped by distance to stimulation site (proximal top, distal bottom). *F*: Peak of subthreshold PSP across muscles cells (deviation from the resting potential) plotted against spike width of a random neuron in the nearest ring segment. Download Figure 5-1, TIF file.

Since the SMN does not consist of a single ring but rather a set of 4 to 13 parallel running fibers of giant neurons per cross section (Singla, 1978; Spencer, 1979), we here model not just a single ring of neurons but rather a set of 12 rings, within a radius of 1.5 cm, that are interconnected randomly at various points. In each ring, the neurons are gap junction coupled to their two closest neighbor, forming a full loop. These rings are stacked on top of each other and connections between neighboring rings are randomly distributed. Each neuron on the ring also has a probability to form a chemical synapse to the closest muscle cell, which are arranged in concentric rings in which each muscle cell is electrically coupled with all boardering cells (see Fig. 2). This means that multiple neurons innervate a specific muscle section. We use this setup to test if synchronous firing of the neurons innervating a particular section can explain the negatively correlated relationship between spike width and PSP amplitude. Because the distance between recording electrodes on neurons and muscles was inconsistent in the original experiments (Spencer, 1982) we here use very thin muscle compartments, with a width of 30 μm and stochastically choose a compartement to measure from, to quantitatively match this variability in the measuring distance from the nerve ring, which influences the PSP amplitude strongly, because measurements taken in closer proximity to the nerve ring will produce stronger PSPs.

In this simulation, a section of the outer layer of the ring is stimulated with a series of postsynaptic currents. After a neuron has spiked, the signal travels around the ring in both directions, until the two action potentials cancel out on the opposing end. Each neuron (that is connected to a muscle) is presynaptic to the nearest muscle segment. Both muscle and neuron voltage traces of one half of the ring can be found in Figure 5*C* & *E*. As in the strip model (Fig. 4), the neuronal spikes in the ring model shorten with distance from the stimulation site. Those spikes depolarize postsynaptic muscle cells. Muscle PSP amplitudes increase, and the delay between neuron and muscle spiking decreases, with distance from the stimulation site (Fig. 5*D*) in agreement with experimental observations (Spencer, 1982). Gap junctions between parallel running fibers were distributed randomly (see Methods). As a consequence, timing of neuronal spikes close to the stimulation site remains variable across cells. As the signal travels along the ring, however, the activity across parallel neuron fibers locally synchronizes via their gap junctions (Fig. 5*D*) and the inputs to muscle cells further down the ring are hence more confined in time (muscles cells each receive synaptic inputs from several, progressively synchronized neuron fibres). The resulting muscle PSPs, therefore, tend to sharpen and increase in amplitude as activity travels along the ring (Fig. 5*B*), until, eventually, PSPs start to surpass the muscle cells’ spiking threshold (at a ring position that is distinct from the stimulation site). Interestingly, once a muscle cell spikes, this mechanism causes a backward spread of activity in the muscle cell population, traveling from the site of the first muscle spike back to the stimulation site (due to the electrical coupling among muscle cells). Initially, the respective muscle cells are not able to spike via the synaptic input of the SMN neurons during the forward spread of activity (due to the larger neuronal spike timing variability the resulting muscular PSP is not large enough to trigger a spike in the muscle cell, see Fig. 5*E*); these cells initially remain silent. Firing of muscle cells close to the stimulation site is thus muscle-cell initiated (on the backward wave) and not immediately triggered by the forward SMN input. Consequently, close to the stimulation site, the delay between SMN-induced PSPs and spike times in muscle cells is particularly large. This creates a strong correlation between the local variability of neuronal spike times and the delay between neuronal and muscular firing (Fig. 5*D*, also compare Fig. 5*E* *top* with Fig. 5*E* *bottom*).

Taken together, along the ring neuronal spike widths shorten and muscle PSPs amplitudes increase. The model thus reproduces a negative correlation between neuronal spike width and muscle PSP, similar to the experimental observations of Spencer (1982) (Fig. 5*F*). Contrary to previously hypothesized mechanisms, however, these two observations need not be causally related. Our model analysis shows that the potassium-channel-mediated reduction of spike width along the ring arises independently from the gap-junction-related increase in synchrony. In our model, each presynaptic spike triggers a stereotyped PSP in the muscle cell once a given presynaptic voltage threshold is surpassed; the effect on the postsynaptic cell does hence per definition not depend on spike width. To illustrate and verify this, we repeat the simulation with a singular strong stimulus, which elicits SMN firing but does not produce wide spikes (Extended Data Fig. 5-1). In this scenario the spike width - PSP anticorrelation is lost, while the pattern of muscle spiking and the correlation between spike time variability and muscle delay remain. While we, depending on the biophysics of synaptic transmission, cannot generally rule out a certain contribution of spike width to PSP size in the jellyfish, our model demonstrates that the previously suggested direct mechanistic link between spike width and PSP amplitude (Spencer, 1982) is not required to explain the dominant part of the negative correlation.

### Spike synchronization increases the speed of muscle contractions

The cascade of backward propagating muscle spikes raises novel questions. Activity propagation via the neuronal fibres is significantly faster than the propagation between muscles (Spencer, 1978). Conduction via the muscle cells, therefore, at first glance, seems ineffective, and a spike in each muscle cell directly induced from its neuronal inputs is likely to be faster than muscle propagation on its own, thus benefiting the overall speed of the animal’s muscle contraction and increasing swimming efficiency (e.g., the distance traveled per stroke). Therefore, one might expect that neuro-muscular synapses should be sufficiently strong to elicit immediate muscle spikes even with more unsynchronized neuronal input. This would also change the direction of muscle propagation with potential effects on the swimming performance, which we will investigate later, but for now we solely focus on the effects on speed and symmetry of the muscle contraction.

**Figure 6.**
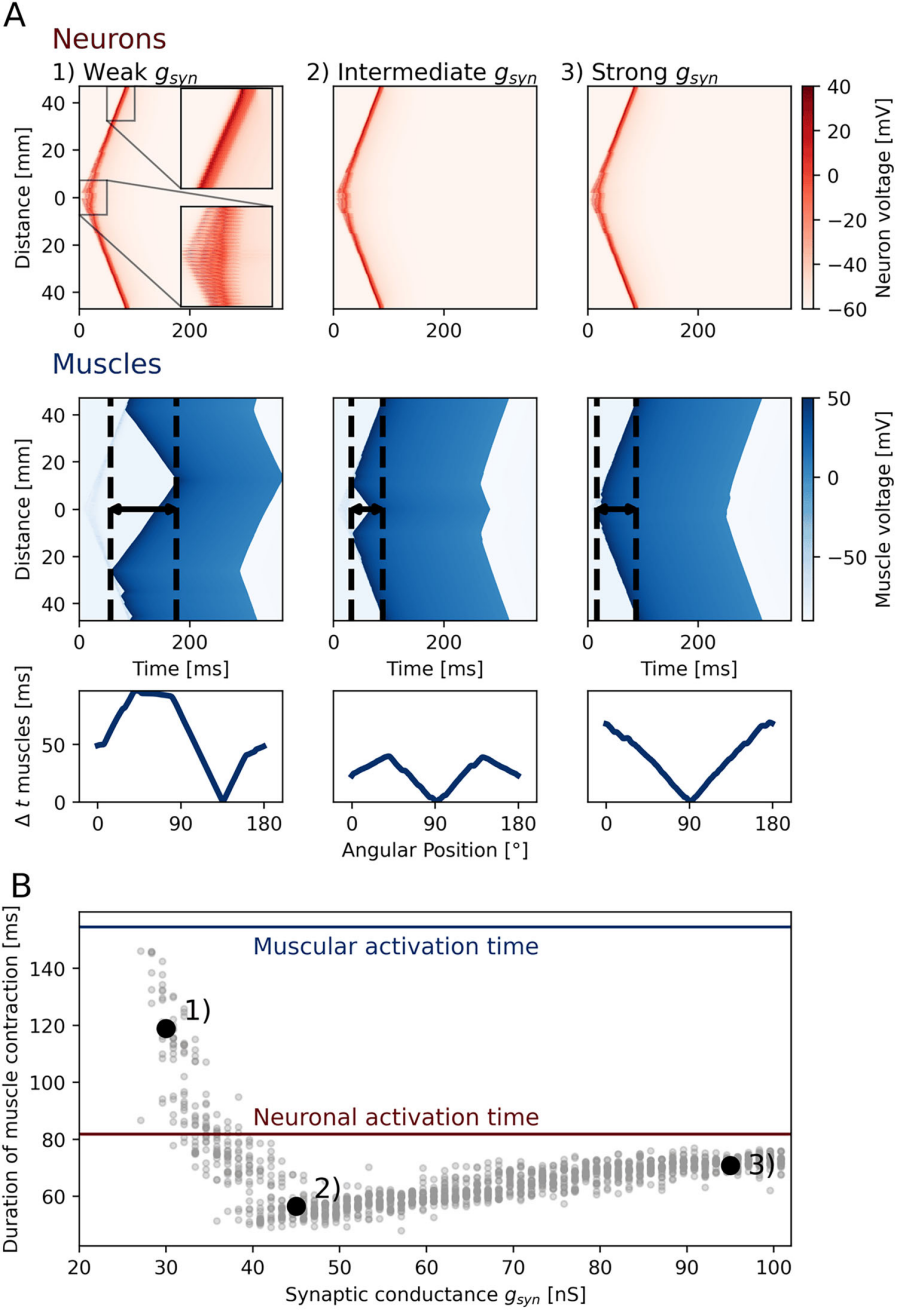
Muscle contraction speed in ring models is maximized at intermediate synaptic strengths. Multiple simulations as seen in Figure 5 with varying synaptic conductance *g*_syn_ (see Eq. 7a) in the neuro-muscular synapse are tested for the resulting total muscle contraction time. ***A***, Example simulations for three different values of synaptic conductance. Voltages of neurons (red) and muscles (blue) are color-coded as a function of time (horizontal) and position along the bell (vertical, distance to stimulation site is indicated). Arrows indicate the duration of the full muscle contraction. The bottom plot shows the time difference in firing between opposing muscle cells as a function of their position along the ring. Lower values indicate more synchronous firing. ***B***, The time it takes for all muscle cells in the ring to spike (temporal difference between first and last muscle spike) as a function of the strength of the neuro-muscular synapse. Red and blue lines indicate the time that would be needed for a full purely neuronal and purely muscular propagation (if stimulated in isolation at a singular point).

We therefore investigate the influence of neuromuscular synapse strength on the overall muscle contraction speed. We model the ring section with a radius of 1.5cm as before, stimulating a section at the margin of the ring to induce an activation wave. By increasing the synaptic conductance of the neuromuscular synapses, we find that we can alter the duration of a full muscle contraction (the time from the first spiking muscle until all other muscles have spiked, see Fig. 6). The range of neuromuscular synaptic strengths explored is chosen such that at the high end, neuronal spikes immediately lead to muscle firing close to the stimulation site, and such that at the low end, the first muscle spike is only triggered at the opposing site of the ring (provided a sufficiently large number of neuromuscular synapses is present at this site). In the latter case, the ensuing propagation of muscle firing exclusively relies on electrical coupling between muscle cells.

Analysing the signal spread around the ring (Fig. 6), we find that intermediate synaptic strengths result in a significantly faster full muscle activation (contraction time <50 ms) than neuronal propagation alone (large synaptic strengths, contraction time 82 ms) or pure muscle propagation (low synaptic strengths, contraction time 155 ms). This effect can be explained as follows: The position on the ring where the first muscle cell initiates a spike due to SMN input, depends on the neuro-muscular synaptic strength. For stronger coupling, spiking is reached at an earlier point, closer to stimulation site. For weaker coupling, this point moves towards the opposite side of the ring. From this point onwards, one can observe two activity waves: the forward-directed muscle activity directly triggered by suprathreshold SMN (traveling towards the opposing end of the ring at the speed of neuronal activity propagation) and the simultaneous backward-directed muscle activity arising from propagation within the muscle sheet via electrical coupling (traveling back towards the stimulation site at the speed of muscular activity propagation). The total muscle contraction time is determined by the time it takes the last of the two waves to reach “its” end of the ring, i.e., the opposing end and the stimulation cite, respectively. If the propagation speed via muscles and neurons was identical, the first muscle spike would occur halfway down the ring and the duration of the muscle contraction could thus be halved in comparison the slower scenario of a propagation from stimulation site to the opposing end. Because the neural propagation speed is almost twice as fast as the muscular propagation, however, the ideal first muscle spike is positioned approximately one third along the way from stimulation site to the opposing end. The duration of the full contraction can for this case be predicted to be at least 33% faster than a direct triggering of muscles via SMN neurons (i.e., the case of very strong neuro-muscular coupling). Due to a partial prior depolarization of the muscle cells in the forward direction, the backpropagation wave can be expected to be slightly faster (than a propagation via completely unstimulated muscle cells). Indeed, in the model we observe a contraction time reduction of 40% (Fig. 6*B*). The model thus provides an alternative mechanism of muscle control, which allows *P. penicillatus* to increase the speed of its muscle contraction beyond what a simple through-conducting wave could achieve. The resulting activation sequence of nerves and muscles is consistent with previous electrophysiological observations, specifically, the variation in delays and muscle PSPs (see Fig. 5*D* & *F*).

In addition to the shortening of contraction time, an intermediate synaptic conduction also results in a more symmetrical muscle activation wave. The sequence of muscle activation becomes more point symmetric when viewed from the center of the jellyfish, i.e., if two opposing points are chosen on the ring, the corresponding muscle cells fire more closely in time for intermediate synaptic strengths than they would for very weak or very strong synapses (Fig. 6*A* *bottom*). We propose that this increased symmetry aids in straight swimming, whereas strong delays in muscle activation time could lead to an imbalanced swimming motion and would turn the jellyfish. Moreover, due to the nature of the electrical coupling of muscle cells, muscle spike widths vary as well, with longer initial spikes and shorter spikes later on (see for example Figs. 5*B* and 6*A*). Consequently, their is more temporal overlap between the activation of the population of muscle cells, effectively also reducing the time spent on muscle deactivation. Experiments have demonstrated such spike shortening in the epitheliomuscular cells and it has been hypothesized that these changes in muscle spike width might aid in synchronization (Spencer and Satterlie, 1981).

### A model of the jellyfish subumbrella creates fast, symmetric muscle activation

The SMN neurons only interact with the muscle sheet locally when they fire and the circular muscles are able to conduct activity on their own. To fully capture muscle activation across a complete SMN, the circular ring model needs to be expanded towards the center of the animal. For the muscular sheet, this means the addition of concentric muscle rings of progressively smaller radius until the animal’s center is reached. The neuronal connection of these sheets is achieved via the four radial nerves extending from the outer nerve ring (we note that, anatomically, it is termed the inner nerve ring, see Fig. 3) towards the center of the jellyfish, where they meet in a small circular section around the manubrium. The circular muscle sheets are gap-junction connected both circularly and radially, taking into account that the radial connection is significantly weaker in accordance with experimental observations (see Methods). This setup essentially creates four separate muscle sections that are surrounded by SMN neurons.

**Figure 7.**
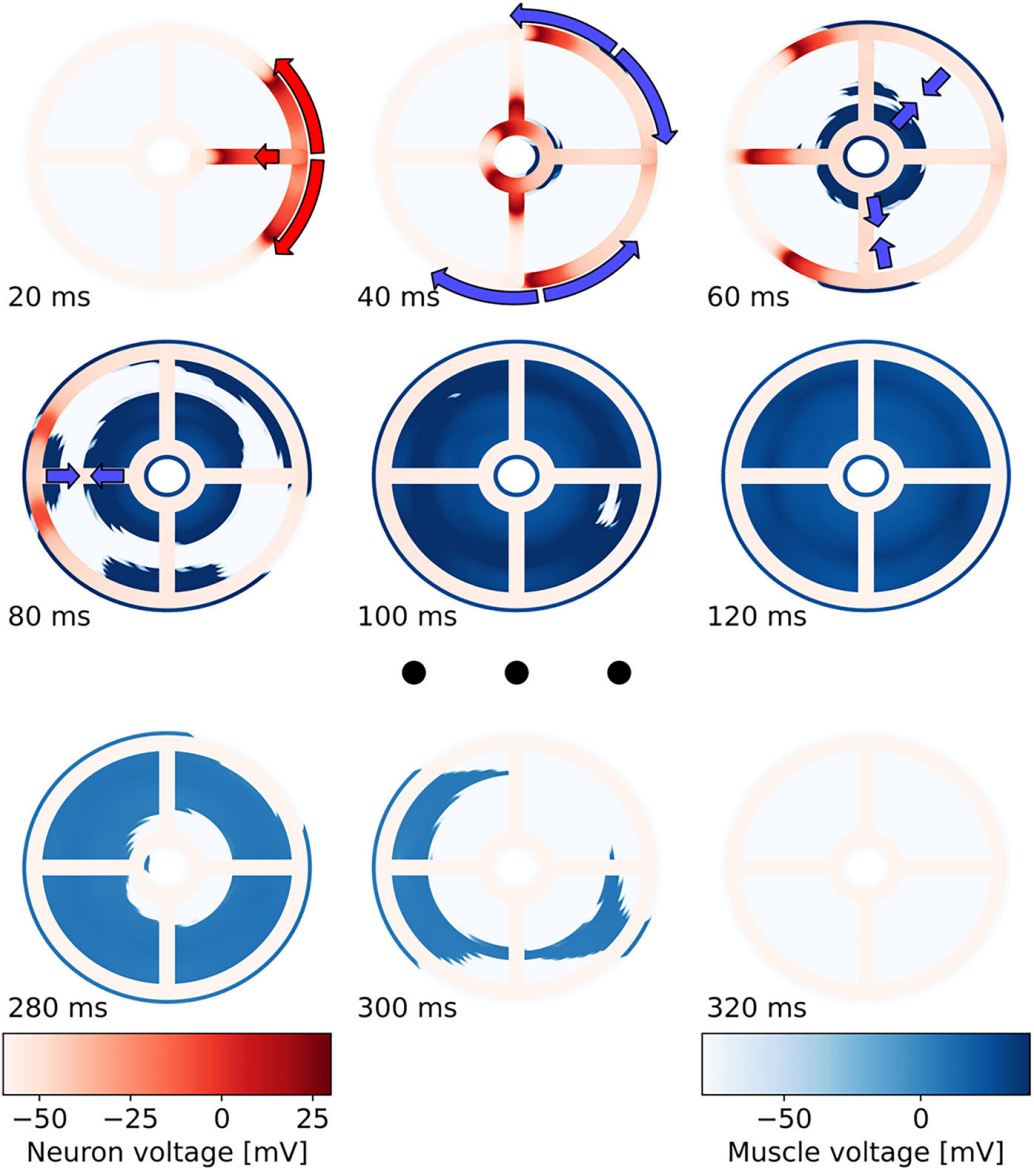
Temporal evolution of muscle activation patterns in a 2D model of the *P. penicillatus* subumbrella. Color-coded voltage of neurons and muscle cells in the subumbrella model, respectively, for different points in time (relative to stimulation). Neurons were stimulated at the right side of a 2D subumbrella model with a radius of 1.5 cm. Arrows indicate the direction of neuromuscular activity propagation. Along the margin, the muscle activity propagates as described in Figure 6. Muscles, however, also fire in the center of the subumbrella (due to neuronal activiation), creating two wavefronts that meet halfway.

Stimulation of a small region of neurons of the inner nerve ring leads to the spread of neural activity and soon after to the spiking of muscle cells around the margins of the bell. The neural activity then propagates further along the radial neuron strips, and along the manubrial nerve ring (Fig. 7). Similarly to the ring model simulations, muscle activation on the opposing ends of the jellyfish is almost simultaneous. Notably, the muscle activity propagates both from the margin of the bell as well as from the center. The latter is likely important for efficient jet propulsion as water needs to be rapidly ejected from the bell, for which a sequential activation of the muscles from the margin towards the center may be sub-optimal (Dabiri et al., 2006). Contrary to what has previously been proposed (Spencer and Arkett, 1984), initiation of muscle firing at the radial canals is rare in the model, at least when other parameters are kept unchanged, because PSPs cannot build up along radially neighboring muscle cells due to the reduced gap junction strength. Also, because circularly neighboring muscle cells are not postsynaptic to any SMN neurons, they act as very strong sinks to the depolarization of the muscles along the canal. If the radial canals would produce muscle firing more easily, it would be able to create a symmetry breaking firing pattern, depending on where along the ring the SMN was stimulated.

### Muscle synchrony influences swimming performance of hydrozoan jellyfish

**Figure 8.**
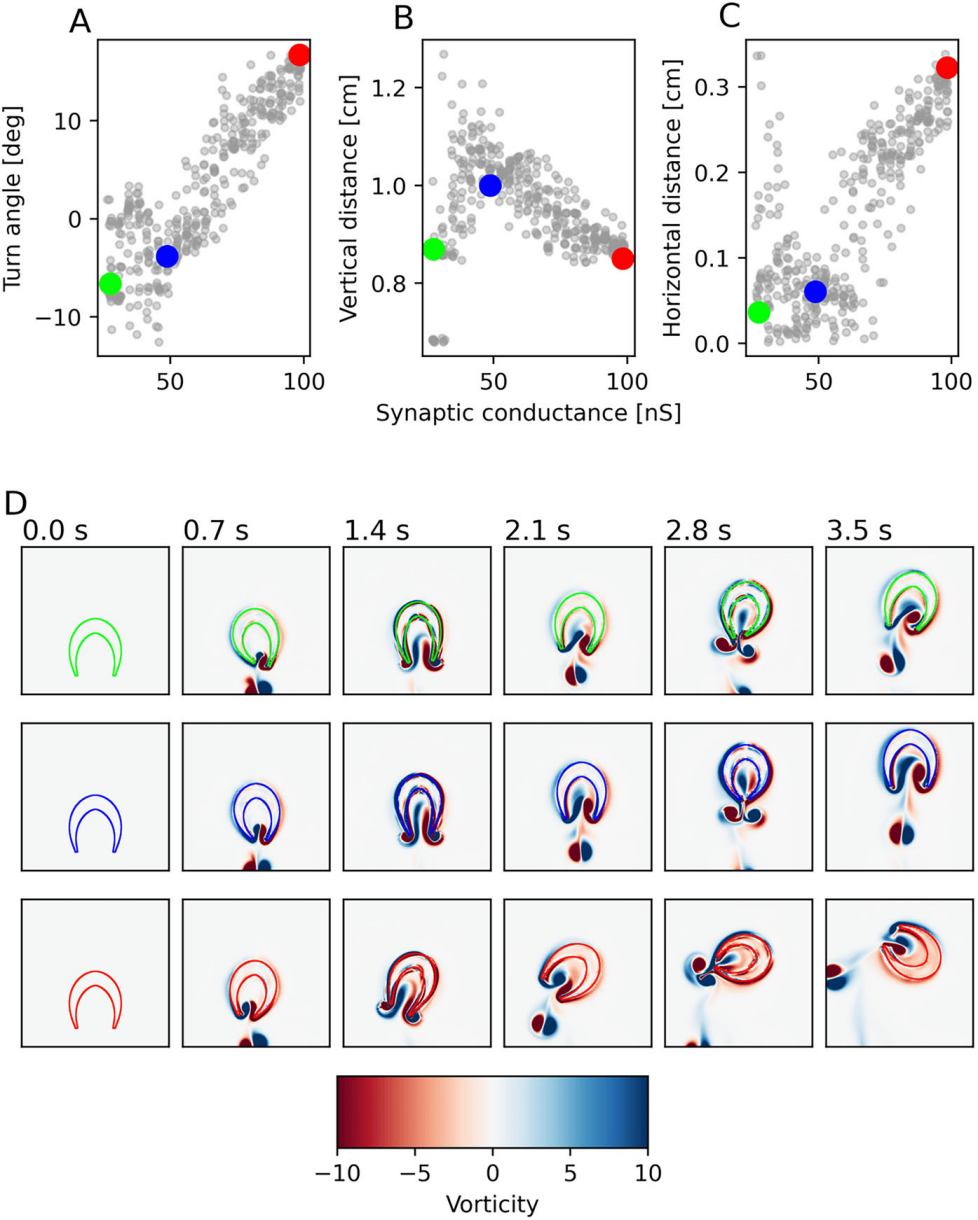
Swimming performance of a 2D hydrodynamics simulation depends on synaptic strength in neuromuscular synapses. Hydrozoan bell models were simulated with varying synaptic strength similar to Figure 6 and the resulting muscle activity was used as input in a 2D hydrodynamics simulation. ***A***–***C***, Three different properties of swimming as a function of the neuro-muscular synaptic strength: The angle by which the jellyfish turned during one swimming stroke (*A*, positive values indicate a turn towards the site of spike initiation), distance traveled vertically (in the indended direction) during one stroke (*B*), distance traveled horizontally during one stroke (*C*). ***D***, Three example simulations of the hydrodynamics simulations for weak, intermediate and strong synapses over the course of three swimming strokes (synaptic strengths and swimming characteristics as indicated by colored circles in *A*–*C*). Swimming strokes were preformed with an interval of 1.2 s between them. Fluid coloration describes the vorticity, with red corresponding to a clockwise and blue to an anti-clockwise vortex.

To estimate the robustness and sensitivity of swimming motion to asymmetries in activation patterns, we conducted a series of swimming simulations in a two-dimensional fluid-structure simulation. To this end, we first developed a 3-dimensional version of our ring model by stretching the 2D model over the 2D crossection of the bell (see Methods) to get more accurate distance measures along the neuromuscular sheet and simulated this cross-sectional model of the jellyfish bell in fluid using the IB2D package, which has been previously used to model jellyfish swimming (Pallasdies et al., 2019; Baldwin and Battista, 2021; Miles and Battista, 2021). The performance of the individual runs of this simulation were quantified based on, first, how much the jellyfish turned, second, how far it traveled vertically, and, third, how much it was displaced horizontally after a single swimming stroke (Fig. 8). Ideal results minimize the amount of turning and horizontal displacement, while maximizing the distance traveled vertically.

Interestingly, peak performance across all three measures aligns with the minimal contraction time measured in Figure 6, with an intermediate neuromuscular conductance of 45–55 nS. Angular displacement can be reduced to an even smaller amount than in previous simulations of scyphozoans (Pallasdies et al., 2019). The same is possible for horizontal displacement which in the scyphozoan model has always been on the order of 2 mm, irrespective of the number of neurons used in these simulations. This demonstrates the clear advantage our proposed hydrozoan nerve net dynamics pose over those of the scyphozoan net. The ability to turn an asymmetric stimulus into a symmetric muscle activation pattern is highly important for straight swimming. The reduction in horizontal and angular displacement coincides with a larger vertically traveled distance (Fig. 8*A–C*). Since total muscle output is very similar between different simulations, these measures are all highly correlated, as distance traveled horizontally is distance lost vertically.

In sum, these results support the hypothesis, posed by our nerve ring model, that the parameters of connectivity are such that they allow for minimal delay and maximal symmetry of the muscle activation pattern.

## Discussion

We investigated the swimming motor network (SMN) in hydrozoan jellyfish from the level of neuronal biophysics to behavior. Based on a conductance-based neuron model constrained by electrophysiological data, we reproduce the spike-shortening effect seen in experimental recordings of the SMN, tracing it back to the inactivation of potassium channels. Connecting neurons and muscles in physiologically realistic ring models, we find that the paradoxical negative correlation between spike width and muscle PSP amplitude is explained by an increase of synchrony of firing neurons over a given segment. Accordingly, a previously assumed causal relation between spike width and muscle PSP is not required to explain the experimental observations. We show that the synchronization provides a mechanism by which a symmetric muscle activation pattern can be created from an asymmetric stimulus. Total muscle contraction time is shortened by initiation of the muscle contraction at a location different from the point of stimulation, temporarily causing a simultaneous spread of muscle activation in four different locations. Fluid-structure simulations demonstrate that the synchronization mechanism improves the swimming performance of hydrozoan jellyfish and is both necessary and sufficient for stable swimming behavior.

### Spike-width shortening

While the model exhibits the experimentally observed spike width shortening, the latter is mechanistically not required for the increase of muscle PSP amplitude over time. Whether it serves a functional purpose remains unclear. The wide neuronal spikes close to the stimulation site could be needed for the extended refractory period mediated by them after spiking. Due to the bi-directionality of jellyfish synapses, a neuron might quickly spike again in response to input from a neighboring neuron if its refractory period was too short. Wider spikes might hence prevent repetitive firing. For neurons further along the ring, the shortened spike width reduces their refractory time, rendering them excitable again directly after the activity wave traveled through the SMN. It remains unclear, however, why this faster reaction time of the SMN would be necessary, as the muscle activity of a swimming stroke is orders of magnitude slower than the SMN. We note that other hydrozoans require multiple quick SMN activation waves to produce a swimming contraction, meaning their SMN neurons would have to fire multiple times in rapid succession for the initiation of muscle spiking (Satterlie, 1985). In addition, a damaged *P.penicillatus* with only a partial nerve ring remaining can often still produce a swimming motion. In our model, multiple quick SMN activation waves producing superimposed muscle PSPs would be required to achieve this.

### Toward an accurate whole-body hydrozoan model

There are two structural considerations that may lead to qualitative differences between our model and a real hydrozoan. First, we simplified the connectivity in the inner nerve ring. We assume a rung-ladder-like connectivity, because that is how connections in the radial nerves have been described, and neurons along the margin have a clear preferentially circular orientation (Lin et al., 2001). The connectivity along the ring, however, seems to be more complex and includes neurons of varying sizes (Singla, 1978; Spencer and Satterlie, 1980; Lin et al., 2001). They connect to each other and to the large motor neurons but their function is unknown. We have not studied how the addition of differently sized neurons would affect the network nor how a more complicated connectivity would influence the dynamics. The synchronization mechanism, however, does not rely on a particular connectivity (as long as synapse placement is heterogeneous), and while quantitative differences to real hydrozoans are likely, the qualitative synchronization effect of the gap-junction-coupled ring is likely to remain.

Second, the connectivity of the radial nerves and their synapse formation with the muscles had to be estimated. Neurons of the radial canals are connected to the inner nerve ring (Singla, 1978; King and Spencer, 1981; Lin et al., 2001), but it has not yet been experimentally quantified how tightly and how many synapses are formed with the circular muscles. We treated the SMN as homogeneous across the nerve ring and the radial nerves. While that assumption is reasonable along the margin of a radially symmetric animal, it may not hold up along the radial nerves and near the manubrium. If this connectivity differed from the marginal nerve ring, then it would affect when and where muscles along the radial nerves and in the center of the jellyfish bell fire, thereby changing the sequence of muscle activation and, potentially, the swimming pattern. Experiments by Spencer and Satterlie (1981), however, suggest that the radial nerves do indeed connect to the circular muscles in a similar way implemented in our model because they observed the initiation of muscle activity along the margins of the four quadrants created by the inner nerve ring and the four radial canals. In principle, our model can be expanded to include future measurements on the neuromuscular connections of the radial nerves. Alternatively, it could be used to generate predictions about their nature, once time-resolved muscle activity data becomes experimentally available.

Moreover, we note that not all neuronal and muscular mechanisms that can influence behavior were exhaustively considered. Specifically, activity of the outer nerve ring was not included. The latter consists of multiple sub-networks that reach down to the tentacles and are responsible for sensory integration and reaction to the environment (Grimmelikhuijzen and Spencer, 1984; Arkett and Spencer, 1986a, 1986b). In addition, epithelial cells are, to an extent, electrically active and gap junction-coupled, providing another system through which hydrozoan jellyfish can sense their environment and alter their behavior (Mackie, 2004). There also seem to exist mechanisms for inhibition and dopamine modulation which are currently not well understood (Chung et al., 1989; Satterlie, 2008; Mackie et al., 2012). Lastly, the radial muscles of the velum, a ring structure around the margin of the bell (see Fig. 3), can contract and direct the ejection of water during a swimming contraction, apparently aiding in the turning of the animal (Gladfelter, 1972; Arkett et al., 1988; Satterlie, 2018). Data on these other electroactive components are scarce, but the model presented here could be readily expanded to a full description of the neuromuscular system, once experimental data on a level of detail comparable to the SMN become available.

Finally, studying differences in propulsion with fluid-interaction simulations in two dimensions provides an approximation to the three-dimensional behavior. Oblate jellyfish, unlike *P. penicillatus*, swim by shedding vortex rings from the margin of the bell and these tend to expand over time (Herschlag and Miller, 2011). In 2D, these vortex rings are reduced to pairs of individual vortices that are able to collapse onto one another. Prolate jellyfish, like *P. penicillatus*, however, swim by using a jetting motion, propelling forward by ejecting water quickly out of the jellyfish bell (Colin and Costello, 2002; Sahin et al., 2009; Weston et al., 2009). This motion can be approximated much better in 2D, and within the frame of this study it allows us to run longer simulations of multiple swimming strokes.

### Toward understanding embodied nervous systems

There has been a recent surge in research interest on cnidarian nervous systems (Vogt, 2022) accompanied by novel modelling efforts. For example, genetic algorithm optimisation of *in silico* simulations of coral nerve nets have found parameter regimes that reproduced experimentally observed spread of excitation (Chen et al., 2008). Furthermore, researchers have been able to record and categorize a stable behavioral repertoire as well as nearly all neurons of *Hydra vulgaris*, bringing the idea of a holistic understanding of a nervous system model within arm’s reach (Dupre and Yuste, 2017; Han et al., 2018). Wang et al. (2023) have implemented insights from these experiments into a multi-scale model of parts of the neuromuscular system which is able to reproduce some of *H. vulgaris*’s behaviors.

We here provide an example of how embodiment influenced nervous system structure and dynamics, emphasizing the point of view that taking the physical body into consideration can be a crucial step for understanding nervous systems. Moreover, the comparison of our hydrozoan results with the previous work on scyphozoan jellyfish (Pallasdies et al., 2019) highlights how two related species evolved anatomically and mechanistically different neural structures to produce similar behavior in tandem with their respective body morphology. Mathematical models of different clades and developmental stages of medusae, in particular, provide the methodology to answer comparative, evolutionary and developmental questions that cannot be addressed with experimental approaches alone. For instance, some hydrozoans alter their bell shape during development (Weston et al., 2009). Studying these animals from the perspective of changes in their neuronal development during the medusan stage could provide interesting insights into the interconnectedness of bell morphology and nerve net structure (Satterlie, 2018). In summary, taking into account recent advances in 3D simulations of jellyfish swimming (Hoover and Miller, 2015; Hoover et al., 2017, 2021), the simulation of complete animals with all their neurons, for jellyfish, seems feasible in the near future (Vogt, 2022). A promising outlook, given that such approaches can help us gain a new level of mechanistic understanding of the evolution of neurons and neuromuscular systems.
