# Supplementary figures and images for: Neuronal Synchronization and Bidirectional Activity Spread Explain Efficient Swimming in a Whole-Body Model of Hydrozoan Jellyfish
Source: J Neurosci. 2025 Apr 9;45(20):e1370242025. doi: 10.1523/JNEUROSCI.1370-24.2025 (PMC12079735; doi:10.1523/JNEUROSCI.1370-24.2025)

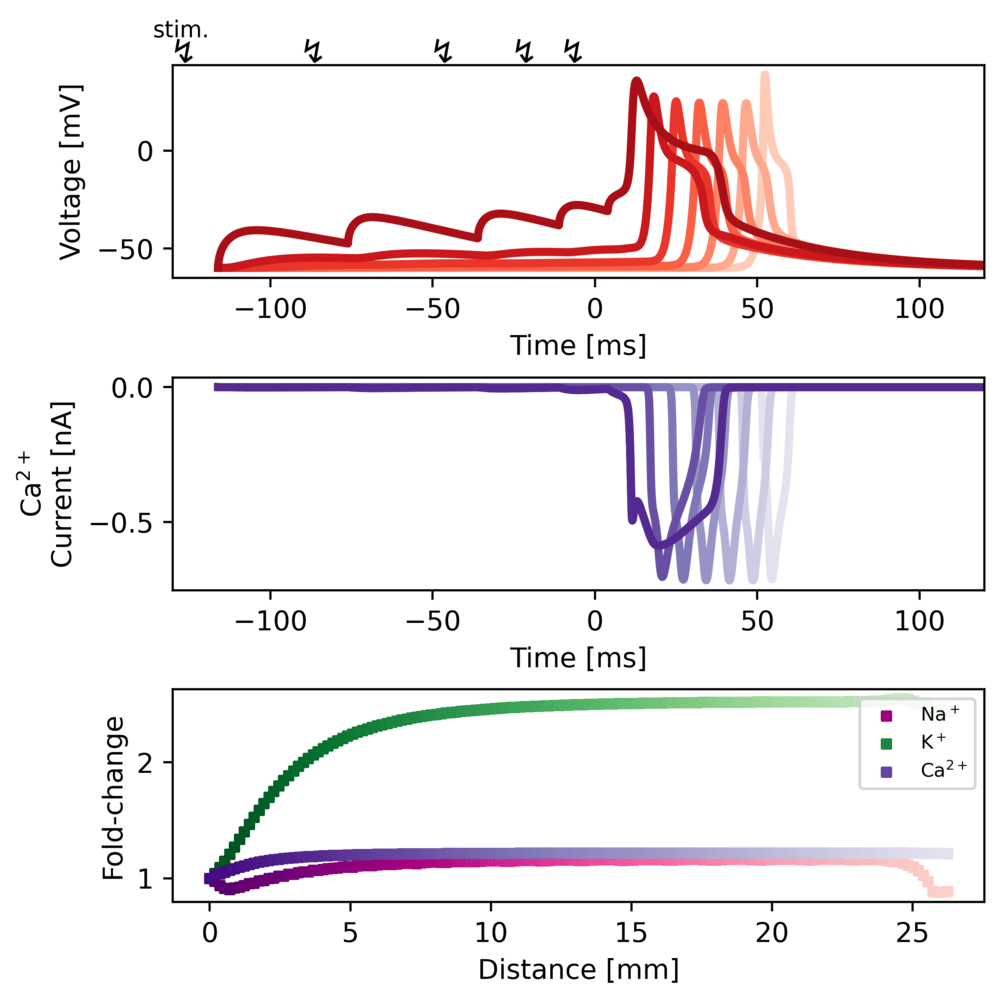

Supplement: Figure 4-1 — Spike shortening in a strip of SMN neurons does not meaningfully change calcium current levels. The simulation setup is identical to Fig. 4 in the main text but here calcium currents are displayed. Top: The voltage measured in different neurons along the strip. Middle: Ionic currents evoked by calcium channels. Bottom: Fold-change of the absolute peak calcium currents along the strip compared to the fold-change of sodium and potassium currents. Similar to sodium, the calcium currents do not meaningfully change along the strip with spike shortening. Download Figure 4-1, TIF file. [file jneuro-45-e1370242025-s001.tif]

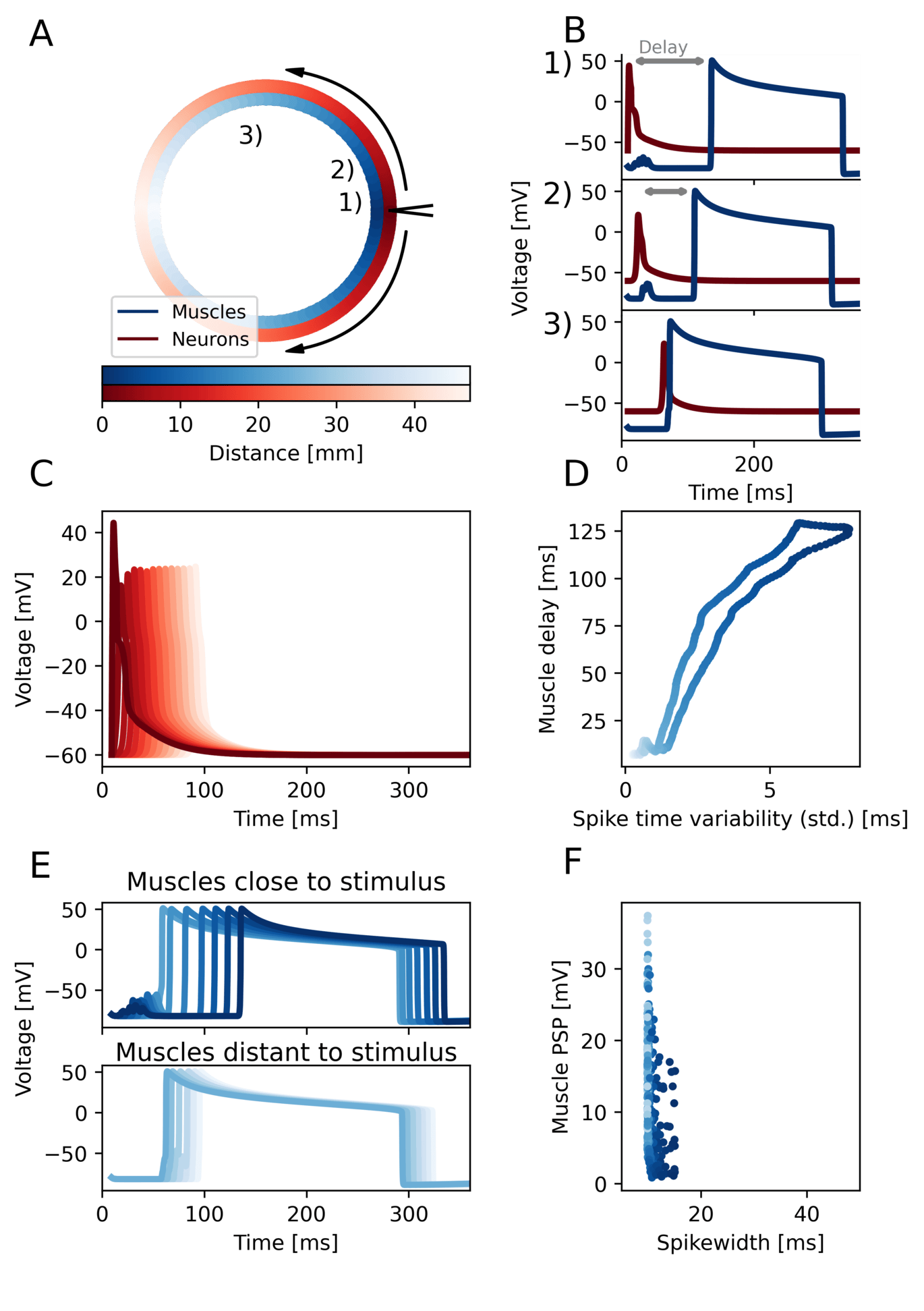

Supplement: Figure 5-1 — Spike shortening is not necessary for synchronization-based spiking. Similar model setup to Fig. 5 but the SMN is stimulated via a singular, short input current, which in effect does not produce wide spikes. This destroys the anticorrelation between spike widths and muscle PSPs but retains all other features of our model, including the pattern of muscle activation and the correlation between spike time variability and muscle delay, thus demonstrating that spike shortening is not causal to the varying muscle PSPs in our model. A: An overview of the ring model. B: Voltage traces of neurons and muscles at three different points along the ring. Grey bars indicate the delay between neuron and muscle spike at the respective location. C: Neuronal voltage traces along the ring; brightness indicates the distance to the site of initiation. D: Spike time variability of the neurons at a local section of the ring plotted against the delay between the local neuron and muscle spikes measured at both paths along the ring. E: Muscle voltage traces for different positions along the ring, similar to C; for better distinguishability muscles grouped by distance to stimulation site (proximal top, distal bottom). F: Peak of subthreshold PSP across muscles cells (deviation from the resting potential) plotted against spike width of a random neuron in the nearest ring segment. Download Figure 5-1, TIF file. [file jneuro-45-e1370242025-s002.tif]
